# Severe cervical compressive polydiscopathic myelopathy with features of motor neuron disease: A case report

**DOI:** 10.1002/ccr3.3740

**Published:** 2021-01-09

**Authors:** Vitalie Văcăraș, Elian Hapca, Nicu Cătălin Drăghici, Dafin Fior Mureșanu

**Affiliations:** ^1^ Department of Clinical Neurosciences “Iuliu Hațieganu” University of Medicine and Pharmacy Cluj‐Napoca Romania; ^2^ Neurology Department of Cluj County Emergency Hospital Cluj‐Napoca Romania; ^3^ Centre of Advanced Research Studies "IMOGEN" Institute Cluj‐Napoca Romania

**Keywords:** amyotrophic lateral sclerosis, cervical myelopathy, motor neuron disease, muscular atrophy

## Abstract

Cervical myelopathy is part of ALS mimic syndrome and should be considered in patients with clinical signs of motor neuron disease.

## Graphical abstract

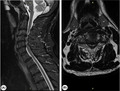

## 1 INTRODUCTION

Cervical myelopathy is a well‐known cause of disability among elderly. It is included in the amyotrophic lateral sclerosis (ALS) mimic syndrome and could resemble motor neuron disease. We present the case of a 70‐year‐old patient with severe cervical compressive polydiscopathic myelopathy with clinical findings of motor neuron disease.

Cervical myelopathy is a widely spread cause of disability among elderly.^1^ The clinical decline is progressive, and this condition should be taken into consideration in patients over 55 years old with loss of motor control of the upper limbs, gait disorders, or sphincter dysfunction. Quality of life could be severely impaired in these patients, evidence showing that beyond the motor, sensory, and bladder dysfunctions recorded with myelopathy scales, there is also impairment of emotional and mental health.^2^

There are several diseases reunited together under the name of amyotrophic lateral sclerosis (ALS) mimic syndrome which can present with a clinical phenotype of motor neuron disease and therefore require to be considered as a differential diagnosis. As ALS is a progressive disease, with disability and with a fatal prognosis, the implications of a misdiagnosis can be of a great importance for patients and their relatives.^3^ In population‐based studies, it is estimated that 8%‐10% of patients referred to a tertiary referral MND (motor neuron disease) center with a diagnosis of ALS will ultimately turn out to have another condition.^4^

At the same time, a recent review describes a number of clinical presentations of ALS with (a) motor neuron involvement (ALS or primary lateral sclerosis, or upper motor neuron predominant ALS, or progressive muscular atrophy, or lower motor neuron predominant ALS); (b) bulbar or spinal onset; (c) focal onset (progressive bulbar palsy, pseudobulbar palsy, flail arm and flail leg); and (d) cognitive involvement (ALS with cognitive impairment and ALS with frontotemporal dementia).^5^

One of the clinical entities of ALS mimic syndrome that can resemble motor neuron disease is cervical myelopathy, which has a better prognosis than ALS and can be alleviated by neurosurgical intervention in most of the cases. Therefore, a clear separation of these entities at the early stage is mandatory and can be a challenge for clinicians.^6^ In addition, degenerative changes of the cervical spine may coexist with motor neuron disease in the elderly, an incidental radiological finding generating great diagnostic difficulties between these two.^7^

There are many other clinically significant mimics of motor neuron disease that could be discussed according to the progression of the disease, the predominant upper or lower motor neuron signs, and the extent of the muscular weakness. Of these, besides cervical myelopathy, we mention spinal muscular atrophy (SMA) and cerebrovascular disease.^8^

## 2 CASE REPORT

### 2.1 Patient history

We present the case of a 70‐year‐old male patient, with proximal weakness of the upper limbs for several years, who came to the Neurology Department of our hospital for gradual worsening of the weakness. Two months prior to the presentation, he experienced weakness of the lower limbs with important gait disturbance and frequent falls. The patient had no significant medical history or long‐term treatment.

### 2.2 General clinical examination

**FIGURE 1:**
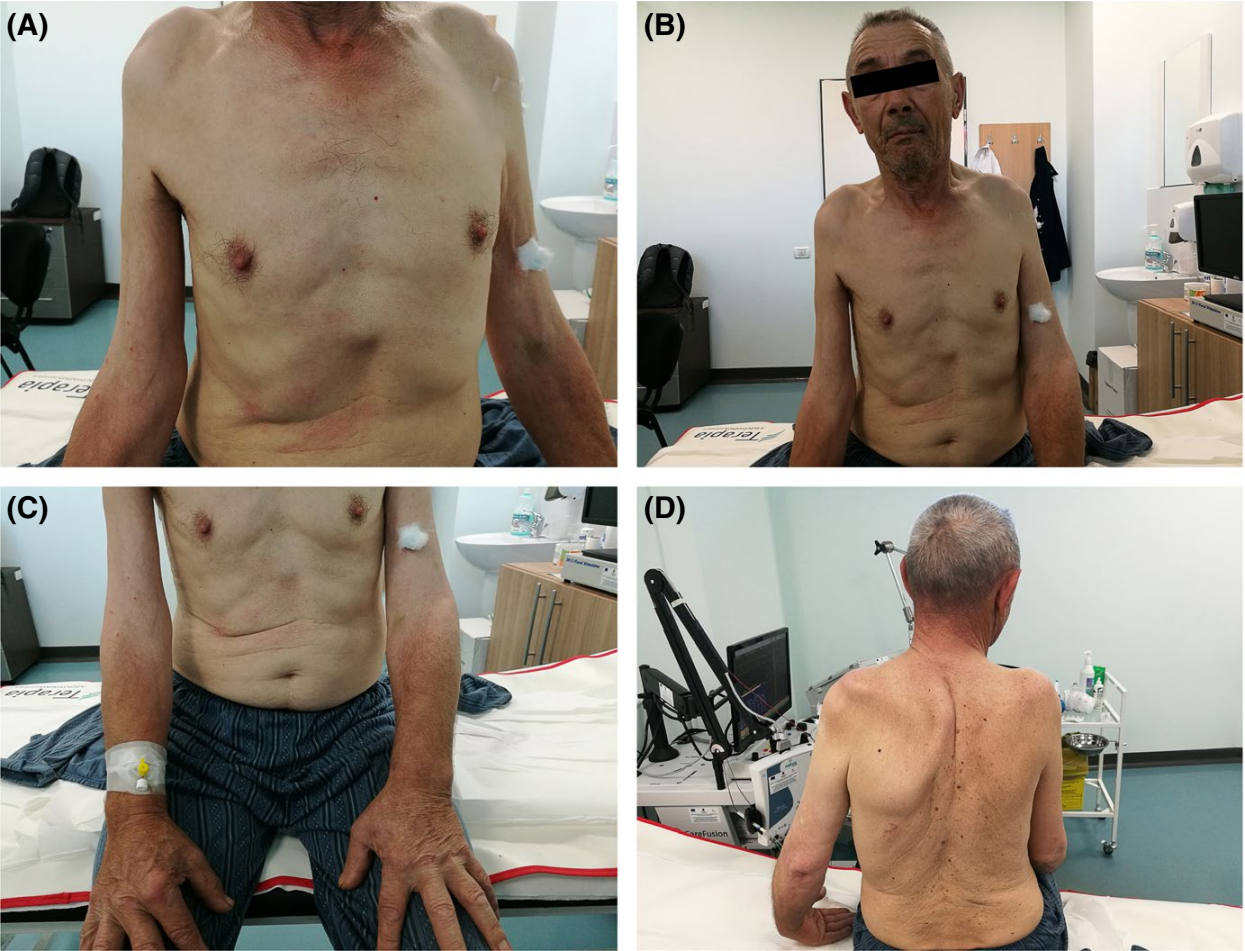
Physical examination findings. A and B, Images showing bilateral muscle atrophy of the shoulder girdle of the patient. C, Images emphasizing the patient's normal aspect of distal muscle mass on upper limbs. D, Kyphoscoliotic aspect of the spine

On the clinical examination (Figure 1), we observed bilateral muscle atrophy of the shoulder girdle, a kyphoscoliotic spine, and varicose veins in the lower extremities. The oxygen saturation level was 98%, blood pressure was 120/85 mm Hg, and the heart rate was 63 beats per minute.

### 2.3 Neurological clinical examination

According to the Medical Research Council Scale (MRC), the neurological clinical examination revealed the following values of muscle strength as presented in Table 1.

**TABLE 1:** MRC muscle strength scale values of the upper and lower limbs

Right MRC	Muscle	Left MRC
0/5	Deltoideus	0/5
3/5	Biceps brachii	3/5
3/5	Triceps brachii	3/5
5/5	Wrist flexors	5/5
5/5	Wrist extensors	5/5
3/5	Hip flexors	3/5
3/5	Knee flexors	3/5
5/5	Ankle dorsiflexion	5/5
5/5	Ankle plantar flexion	5/5

Deep tendon reflexes were generally absent in the upper limbs, but present in the lower limbs.

Abdominal reflexes were depressed on the left side, and the Babinski sign was present bilaterally. There was also a tactile exteroceptive hypoesthesia in the upper left limb with occasional paresthesias, and also urinary incontinence. Moreover, the patient described muscle fasciculations of the upper limbs in the recent history. Cranial nerves were normal, and no family history of neurodegenerative disorders was described.

### 2.4 Nerve conduction studies

To rule out a peripheral nervous system pathology, we performed nerve conduction studies (NCS) in the upper and lower limbs, with normal parameters (latency, amplitude, motor and sensory nerve conduction velocity) for all the nerves (Table 2).

**TABLE 2:** Electrodiagnostic studies

Study	Nerve	LAT (ms)	AMP (mV)	CV (m/s)
Motor	Ulnar.L	Wrist—3.3 Below elbow—7.1 Above elbow—9.3	Wrist—10.7 Below elbow—9.7 Above elbow—9.9	Below elbow—58 Above elbow—45
	Median.L	Wrist—4.2 Elbow—8.3	Wrist—4.4 Elbow—4.4	Elbow—51
	Tibial.L	Ankle—4.9 Popliteal fossa—13.7	Ankle—14.0 Popliteal fossa—11.3	Popliteal fossa—41
	Peroneal.L	Ankle—3.5 Fibula (head)—10.0 Popliteal fossa—12.5	Ankle—8.6 Fibula (head)—8.5 Popliteal fossa—7.8	Fibula (head)—46 Popliteal fossa—40
Sensory	Ulnar (wrist).L	Onset—2.6 Peak—3.6	13 µV	40
	Median (digit II—index finger).L	Onset—3.1 Peak—4.8	19 µV	41
	Sural (lower leg).L	Onset—3.4 Peak—4.3	12 µV	40
	Sural (lower leg).R	Onset—3.6 Peak—4.5	8 µV	41

*F*‐wave studies	M‐Latency	F‐Latency
Ulnar.L	3.2	28.4
Median.L	4.3	29.0
Tibial.L	4.4	51.9

Abbreviations: µV, microvolts; AMP, amplitude; CV, conduction velocity; L, left; LAT, latency; m/s, meters/second; ms, milliseconds; R, right.

### 2.5 Needle electromyography

A needle EMG (electromyography) was also performed in bilateral biceps brahii, left deltoideus, brachioradialis, first bilateral dorsal interossei, vastus lateralis, and anterior left tibial muscles suggesting a chronic, slow evolutive neurogenic pattern.

### 2.6 Imaging results

**FIGURE 2:**
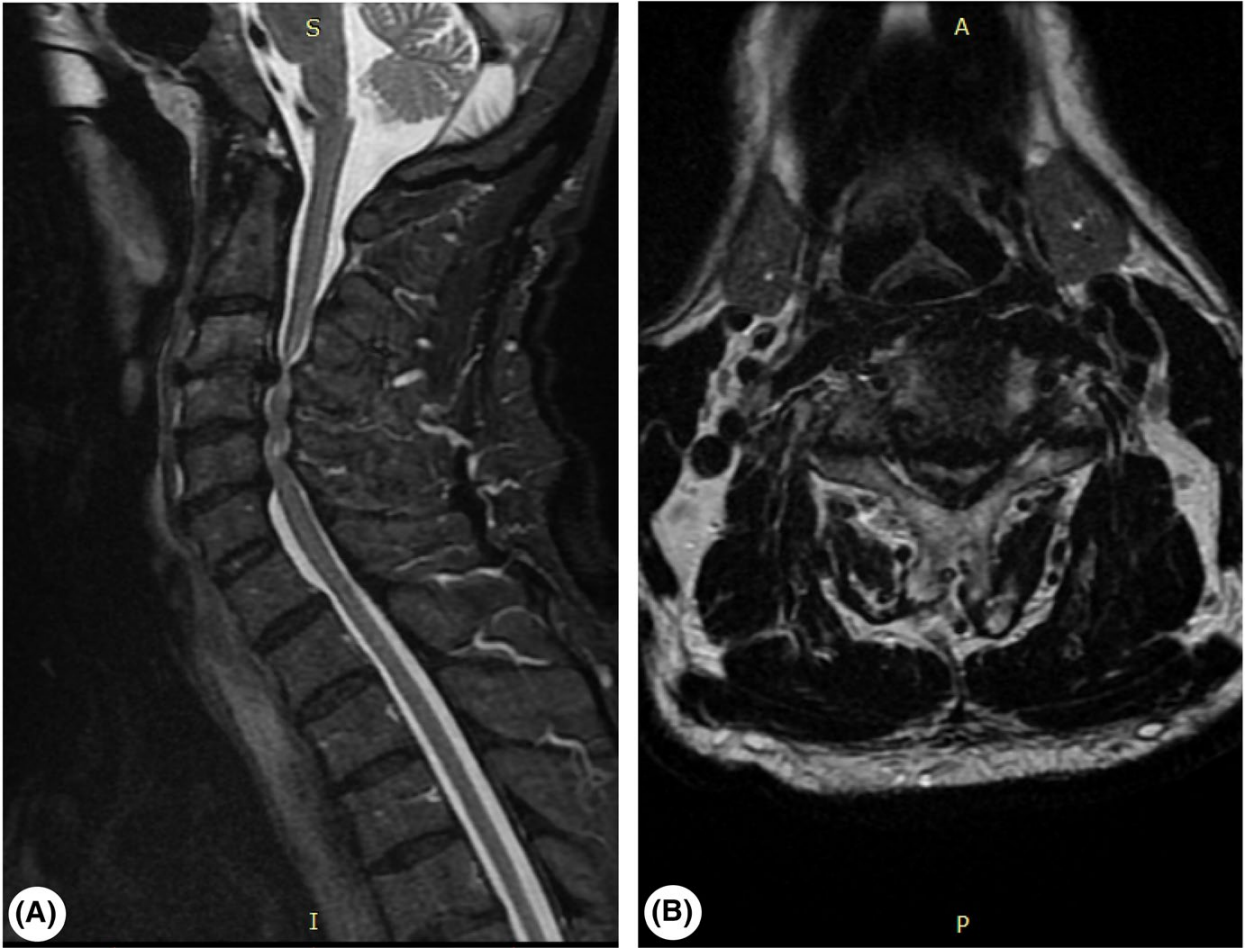
Preoperative cervical nonenhanced MRI aspect. A, Sagittal STIR sequence, demonstrating significant compressive spinal cord suffering from multiple disk herniations and severe stenosis of the spinal canal. B, Axial T2‐weighted frFSE sequence, revealing posterior herniation of the cervical disk, compressing both the spinal cord and spinal nerve roots

Considering the patient's clinical signs, phenotype, age and because cervical myelopathy is widely spread among elderly, being one of the most important ALS mimics, we performed a cervical spine MRI. The nonenhanced MRI (Figure 2) demonstrated the following: C2‐C3 circumferential disk protrusion, without obvious dural or root conflict, C3‐C4 posterior disk hernia, approximately 8 mm, with significant compressive spinal cord suffering and bilateral root conflict and stenosis of the spinal canal of about 70%, C4‐C5 left posterolateral intraforaminal and extraforaminal disk herniation, of about 5 mm, with dural contact, left root conflict and spinal canal stenosis of about 40%, C5‐C6 circumferential disk protrusion, with dural contact, bilateral root conflict, spinal canal stenosis of about 50%, and microinjuries associated with diffuse spinal edema in the C2‐C5 segment.

A head CT was also performed for the exclusion of brain lesions describing cerebral atrophy, with no other pathological findings.

### 2.7 Laboratory results

The laboratory studies showed hypoproteinemia (6.31 mg/dL) and hypoalbuminemia (3.49 g/dL) with no other significant findings. A urine culture was also performed to exclude a urinary tract infection, with a negative result.

### 2.8 Positive diagnosis

Based on the electrodiagnostic (EDX) studies and MRI, a positive diagnosis of severe polydiscopathic compressive cervical myelopathy was confirmed.

## 3 DIFFERENTIAL DIAGNOSIS

Given the presence of both upper and lower motor neuron signs, along with the clinical phenotype of the patient, we have considered some of the ALS mimics encountered in the clinical practice.^4^, ^8^ Those disorders were excluded by the paraclinical investigations or other clinical features.

1. ALS, with its several forms of clinical presentation,^5^ is the first differential diagnosis taken into consideration. Of the multiple forms, flail arm syndrome was the most important in our case because of the clinical features that resemble our patient's phenotype. According to the diagnostic criteria for ALS,^9^ this case fulfills the following: presence of upper and lower motor neuron signs, progression of symptoms and signs, neurogenic changes in EMG, and absence of conduction block. However, we excluded this diagnosis because of the presence of an ALS mimic (severe cervical myelopathy) along with other exclusion criteria such as sensory signs and sphincter disturbances.
2. Spinal muscular atrophy, included in the ALS mimic syndrome, was also a possible diagnosis, specifically type 4 with adult onset. The genetic component of this disease consists of homozygous deletions in the SMN1 (survival motor neuron) gene with the presence of three or four copies of SMN2 gene in SMA type 4, making this form a milder one.^10^ This diagnosis was excluded by genetic testing and also by the absence of cases in family history.
3. Despite not being included in the spectrum of ALS mimics, we have considered limb‐girdle muscular dystrophies as differential diagnosis in relation to the clinical appearance of our patient. These are rare conditions with a clinical expression characterized by weakness and wasting of pelvic and shoulder girdle muscles with autosomal dominant or recessive inheritance. There are more than 30 genetic forms recognized, some with adult or late‐adult onset.^11^ The clinical phenotype of the patient could fit the definition, but the needle EMG showed a neurogenic pattern, serum creatine kinase was normal, and no family history of muscular dystrophies was present, making this diagnosis improbable.
4. Cerebrovascular disease was also considered in our case as studies show that in some populations it is an important ALS mimic.^8^ We performed a brain CT examination that did not reveal any signs of cerebrovascular disease so this diagnosis was also excluded.

## 4 OUTCOME AND FOLLOW‐UP

According to the paraclinical findings, a neurosurgical examination was requested, and with the agreement of the patient and family, a decompressive neurosurgical intervention was performed to decompress the dural sac by C3, C4, C5 laminectomy and posterior rachisynthesis with 4 transarticular screws at C3, C5 level and 2 titanium bars.

After the surgery, the patient was evaluated by a kinetotherapist who started a recovery program tailored for our patient, with active and passive mobilization of the limbs. A hard cervical collar for the neck immobilization was applied. Also, he was progressively raised on the bedside with slow initiation of gait, with a favorable evolution on discharge, and improvement of the symptomatology at 1‐month follow‐up.

**FIGURE 3:**
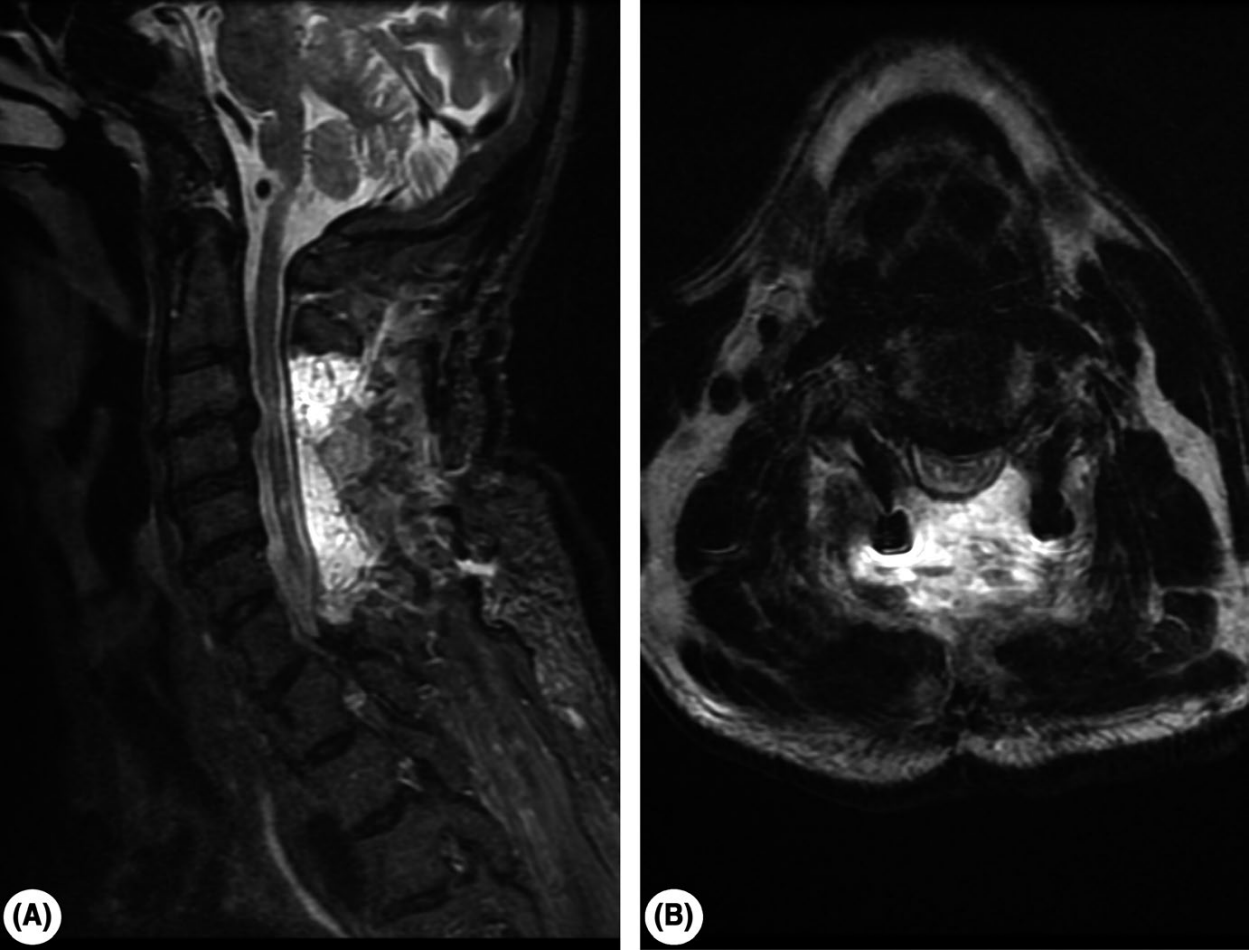
Postoperative cervical nonenhanced MRI aspect. A, Sagittal STIR sequence showing decompression of the spinal cord through laminectomy, with posterior postoperative edema. B, Axial T2‐weighted frFSE sequence, indicating reversal of the conflict on spinal cord and spinal nerve roots, with postoperative edema and artifacts from osteosynthesis materials

The postoperative nonenhanced cervical MRI demonstrated the decompression of the spinal cord, the reversal of spinal nerve root conflicts, and the increase of the diameter of the spinal canal (Figure 3).

## 5 DISCUSSION

This case is complex with a long disease progression pattern and clinical findings that include both upper and lower motor neuron signs. As ALS is a lethal disorder that simultaneously involves both upper motor neuron and lower motor neuron with progression from a region of neuroaxis to others and eventually death from respiratory involvement, it is important to rule out treatable mimics of this fatal disease.^12^

The diagnosis of ALS is based upon clinical criteria that include the presence of upper motor neuron and lower motor neuron signs, progression of disease, and the absence of an alternative explanation (ALS mimics). Although the diagnosis of ALS has improved considerably in recent years,^13^ at present, there is no single test that can confirm or entirely exclude ALS. In clinical practice, the diagnosis is established by the history and physical examination, supported by EDX studies, and not excluded by neuroimaging and laboratory studies.^14^, ^15^ Moreover, the Awaji criteria for the diagnosis of ALS have proved to be of a significant importance in earlier diagnosis and clinical trial entry for this disease, in comparison with the previously accepted gold standard—the revised El Escorial criteria.^16^ In our case, the neuroimaging suggests cervical myelopathy as a possible cause for the clinical presentation. The presence of sensory symptoms and signs, lower motor neuron signs at the level of compression, upper motor neuron signs in lower limbs, sphincter dysfunction, the aspect of the cervical spinal cord on MRI, the electrodiagnostic studies, and absence of cases of neurodegenerative disorders in family support our diagnosis and exclude ALS.

We have also taken into consideration an ALS Regional Variant—Brachial Amyotrophic Diplegia, also known as flail arm syndrome as a differential diagnosis, with motor neuron disease confined to the cervical spinal cord region and the existence of slowly evolving types over the course of many years. The presence of sensory symptoms and signs, the MRI findings, the long evolution of the disease (more than 10 years), and the EDX studies are not supportive for this diagnosis.^17^, ^18^

Spinal muscular atrophy, specifically type 4, as part of the ALS mimic syndrome describes patients with adult onset (>18 years old) that remain ambulant as adults and generally, without need of respiratory assistance. Also, 95% of patients have homozygous deletions in the SMN1 (survival motor neuron 1) gene. Chromosome 5q13 contains two nearly identical SMN genes: SMN1 and SMN2. While loss of SMN1 is essential to the pathogenesis of SMA, the severity of the disease is related to the number of copies of SMN2. Type 4 SMA has either three or four copies of SMN2.^10^ Genetic testing and absence of family cases in the patient's history excluded this diagnosis.

Studies describe cerebrovascular disease as representing up to 9% of the ALS mimics in one population‐based registry, after cervical spondylotic myeloradiculopathy (18.86%) and “MND plus” syndromes (13.2%),^8^ so a proper investigation in this area is mandatory, for the optimal exclusion of diseases mimicking ALS. To further complicate matters, it was described that cerebrovascular injury from different causes (cerebral arteriovenous malformations, stroke – hemorrhagic and ischemic, transient ischemic attack, and subarachnoid hemorrhage) may constitute a risk factor for ALS in the context of a complex model of pathogenesis.^19^ We excluded this possibility by performing a brain CT which showed no signs of cerebrovascular disease.

Being a disease included in the ALS mimic syndrome, along with the findings on clinical examination, cervical myelopathy was also discussed. A nonenhanced cervical MRI was performed, and multiple degenerative discal changes were described with significant compressive spinal cord suffering and bilateral root conflict with approximately 70% stenosis of the spinal canal. Thus, a medullary compressive syndrome was shaped that explained the clinical findings. According to the studies, the natural history of cervical spondylotic myelopathy consists of continued deterioration with the risk of progressive disability and neurological function that never returns to normal.^20^ Also, studies show that surgical therapy is superior to conservative treatments in the case of cervical spondylotic myelopathy.^21^ Nonsurgical management options for patients with mild myelopathy include physical therapy for gait training, occupational therapy for improvement of upper extremity dexterity, and neck immobilization with a hard cervical collar. Patient counseling regarding the hazards of minor cervical trauma and the potential for symptomatic worsening is mandatory in this case.^22^ In our patient, the surgical approach was chosen and the outcome was favorable with improvements of the clinical findings.

## 6 CONCLUSION

Clinicians should take into consideration an ALS mimic syndrome when a motor neuron disease is suspected in a patient. One of the diseases that can mimic motor neuron disease is cervical myelopathy, especially in elderly patients. Thus, a cervical MRI examination must be performed to establish the diagnosis, but NCS and needle EMG are also mandatory for the differential diagnosis.

The particularity of our case is given by the impressive stenosis of the spinal canal, the long evolution of the disease, and the distinctive clinical phenotype of the patient, which could suggest a motor neuron disease. It raises awareness among clinicians that severe cervical myelopathy could mimic an ALS syndrome and needs to be investigated because of the better prognosis and treatment options of the former.

## CONFLICT OF INTEREST

The authors declare that there was no conflict of interest regarding the publication of this paper.

## AUTHOR CONTRIBUTIONS

VV: head of the department, consultant neurologist in charge of the patient, contributed to the conception and design of the work, the acquisition, analysis, and interpretation of the data for the work, revising it critically for important intellectual content, final approval of the version to be published, and agreement to be accountable for all aspects of the work in ensuring that questions related to the accuracy or integrity of any part of the work are appropriately investigated and resolved. EH: contributed to the conception and design of the work, the acquisition and analysis of the data for the work (obtained patient history, performed the clinical examination of the patient, took pictures of the patient), drafting the work, revising it critically for important intellectual content, final approval of the version to be published, and agreement to be accountable for all aspects of the work in ensuring that questions related to the accuracy or integrity of any part of the work are appropriately investigated and resolved. NCD: contributed to the design of the work, the acquisition, analysis, and interpretation of the data for the work (performed the NCS and EMG examinations and interpreted the results), revising it critically for important intellectual content, and agreement to be accountable for all aspects of the work in ensuring that questions related to the accuracy or integrity of any part of the work are appropriately investigated and resolved. DFM: coordinator of the team, contributed to the conception and design of the work, the acquisition, analysis, and interpretation of the data for the work, revising it critically for important intellectual content, final approval of the version to be published, and agreement to be accountable for all aspects of the work in ensuring that questions related to the accuracy or integrity of any part of the work are appropriately investigated and resolved.

## ETHICAL APPROVAL

Ethics committee approval and written informed consent from the patient to publish his case and images of him were obtained.

## Data Availability

Data available on request due to privacy/ethical restrictions.
